# Reducing Unnecessary ‘Admission’ Chest X-rays: An Initiative to Minimize Low-Value Care

**DOI:** 10.7759/cureus.29817

**Published:** 2022-10-01

**Authors:** Lisa Iyeke, Rachel Moss, Rochelle Hall, Jeffrey Wang, Laiba Sandhu, Brendan Appold, Enessa Kalontar, Demetra Menoudakos, Mityanand Ramnarine, Sean P LaVine, Seungjun Ahn, Mark Richman

**Affiliations:** 1 Emergency Department, Northwell Health Long Island Jewish Medical Center, New York, USA; 2 Emergency Department, University of Michigan, Ann Arbor, USA; 3 Emergency Department, Robert Wood Johnson Barnabas Health, Rahway, USA; 4 Internal Medicine Department, Northwell Health Long Island Jewish Medical Center, New York, USA; 5 Biostatistics Unit, Feinstein Institute for Medical Research, Manhasset, USA; 6 Biostatistics Department, University of Florida, Gainesville, USA

**Keywords:** educational intervention, evidence-based guidelines, ed boarding, chest x-ray (cx-ray), high-value care

## Abstract

Introduction

Internal medicine admission services often request a baseline admission chest X-ray (CXR) for patients already admitted to the emergency department (ED) and who are waiting for inpatient beds, despite rarely providing clinical value. Adverse consequences of such CXRs include unnecessary radiation exposure, cost, time, and false positives, which can trigger a diagnostic cascade. Extraneous CXRs performed on already-admitted ED patients can delay inpatient transfer, thereby increasing boarding and crowding, which in turn may affect mortality and satisfaction. In 2016, our ED and internal medicine hospitalist services implemented guidelines (reflecting those of the American College of Radiology) to reduce unnecessary admission CXRs. All relevant providers were educated on the guideline. The primary aim of this study was to determine if there were changes in the percentage of patients with pre-admission and admission CXRs following guideline implementation. Our secondary aim was to determine which patient characteristics predict getting a CXR.

Methods

All ED and internal medicine hospitalist providers were educated once about the guideline. We performed a retrospective analysis of pre- vs. post-guideline data. Patients were included if admitted to the internal medicine service during those timeframes with an admission diagnosis unrelated to the cardiac or pulmonary systems. A CXR performed during ED evaluation prior to the admission disposition time was recorded as “pre-admission,” and if performed after disposition time it was recorded as “admission.” A CXR was "unwarranted" if the admission diagnosis did not suggest a CXR was necessary. The numerator was the number of unnecessary admission CXRs ordered on patients with diagnoses unrelated to the cardiac or pulmonary systems (minus those with a pre-admission CXR); the denominator was the number of such admissions (minus those with a pre-admission CXR). Variables of interest that might influence whether a CXR was ordered were age, gender, respiratory rate ≥20, cardiac- or pulmonary-related chief complaint, ED diagnosis category, or past medical history.

Results

Among admitted patients with diagnoses that did not suggest a CXR was warranted, there was no change in the percentage of admission CXRs (21.7% to 25.6%, p = 0.2678), whereas the percentage with pre-admission CXRs decreased (66.6% to 60.7%, p = 0.0152). This decrease was driven by fewer CXRs being performed on patients whose chief complaint did not suggest one was indicated (p = .0121). In multivariate analysis, risk factors for an unwarranted CXR were age >40 (risk ratio (RR) = 2.9) and past medical history of cardiovascular disease (e.g., myocardial infarction, atrial fibrillation), renal disease, or hyperkalemia.

Conclusion

This educational initiative was not associated with the intended decrease in ordering unwarranted admission CXRs among ED boarding patients, though there was an unanticipated decrease in pre-admission CXRs. This decrease was driven by fewer CXRs being performed on patients whose chief complaint did not suggest one was indicated. Organizations interested in reducing processes with little clinical value might adopt a similar program while emphasizing the lack of benefit to admitted patients through iterative educational programs on hospital admitting services.

## Introduction

In the United States, over 70 million chest X-rays (CXRs) are performed each year [1]. Internal medicine admitting services often request 'admission' CXRs in the belief that each admitted patient needs a baseline admission CXR, despite the American College of Radiology (ACR) [2] and U.S. Food and Drug Administration [3] discouraging CXRs in patients without cardio-pulmonary symptoms. Routine admission CXRs rarely yield clinical value (< 4%), such as changing management or finding previously-unidentified diseases [4-6]. Adverse consequences of unwarranted CXRs include unnecessary radiation exposure, cost, time, and the possibility of generating false positives, which can trigger a diagnostic cascade (computed tomography scan, pulmonology consult, bronchoscopy/biopsy) [7]. Diagnostic radiation has been implicated as a contributor to malignancy; one study found an estimated 700 cases of cancer in the United Kingdom each year were attributable to radiographs [8]. The CXR imaging and professional reading/interpretation costs between $150 [1] to $1,200 [9] per CXR (depending on location); low-income and/or uninsured patients may sustain financial hardship from an unnecessary CXR. Extraneous CXRs performed on already-admitted emergency department (ED) patients can delay inpatient transfer, thereby increasing ED boarding and crowding and potentially affecting mortality and satisfaction [10]. 

To reduce unnecessary healthcare expenditures, the Northwell Health Long Island Jewish Medical Center (LIJ) ED convened a committee focused on value-based care. In 2016, the ED and internal medicine hospitalist services implemented guidelines (Appendix A) reflecting those of the American College of Radiology. The intent of these guidelines was to reduce CXRs ordered in the ED after admission decision if no CXR was warranted on the basis of chief complaint, history/physical exam, or diagnosis. All ED and internal medicine hospitalist providers were educated on the guideline. We studied the impact on the future ordering of CXR for the admission of such patients.

We focused on creating guidelines as a means to decrease CXRs because certain practices continued at our hospital that was inconsistent with updated medical evidence. For example, admission electrocardiograms (EKGs) were ordered commonly on patients without cardio-pulmonary complaints, despite little evidence of value [11]; creatine kinase (CK) and CK-muscle/brain (MB) were routinely ordered in diagnosing acute coronary syndrome, despite troponin being adequate in almost all cases [12]. The guidelines created by this committee were successful in reducing unwarranted admission EKGs [11] and CK/CK-MB testing [12]. Consequently, the committee turned its attention to reducing unnecessary admission CXRs.

The primary aim of this study was to determine if there were changes in the percentage of CXR ordered for patients pre-admission and on admission following the introduction of the guidelines. Our secondary aim was to determine whether certain patient characteristics (demographics, vital signs, ED diagnoses) might predict a pre-admission vs. admission CXR and whether these differed between pre- and post-intervention. We investigated changes in pre-admission CXR rates to determine whether any changes in admission CXR rates reflected a possible influence of ecologic changes in ordering a CXR (i.e., there may have been a general change in CXR ordering rates) rather than the specific impact of this intervention to decrease unnecessary ordering of CXR for admission.

## Materials and methods

In June 2016, LIJ's ED and internal medicine hospitalist services implemented guidelines (Appendix A) to reduce unnecessary CXRs ordered for boarding ED patients. In our ED, providers (ED physicians, physician assistants, or nurse practitioners) order CXRs after performing an in-depth history and physical examination; CXRs are not ordered as part of the triage process. Admitting services (e.g., internal medicine) can request an ED provider to order a CXR on an admitted patient in the ED, but do not order CXRs themselves. Necessity was determined by evaluating the chief complaint, history/physical examination, and diagnosis. The authors educated all ED and hospitalist attending, resident, and mid-level providers once on the guidelines; the venues for this education were the ED daily 'morning briefing' and the hospitalist monthly meeting. We performed a retrospective analysis to evaluate the translation of the guideline into practice, looking at the initiative's impact on future extraneous ordering of CXR for admission. 

Patients admitted to the internal medicine service between July and December 2015 (pre-guideline) vs. July and December 2016 with an admission diagnosis unrelated to the cardiac or pulmonary systems were included in the analysis. To ensure the analysis would include high-volume, relevant conditions, the electronic health record was queried to determine the 20 most-common admission diagnoses. Two authors (MR and SL) independently reviewed these and chose the most common 10 non-cardiac/non-pulmonary diagnoses, which based on experience, would most often not require a CXR as part of the evaluation (Table 1). By including only patients admitted to the internal medicine service for non-surgical management, the authors agreed that diagnoses that might occasionally prompt a CXR (e.g., abdominal pain, intestinal obstruction, or cholecystitis) did not likely warrant one. Patients with similar diagnoses who were admitted to a service other than internal medicine, or who had a CXR as part of their ED workup, were excluded from the analysis.

**Table 1:** Most common admission diagnoses for which CXR was unwarranted, in alphabetical order.

Diagnoses
Abdominal pain
Alcohol/drug abuse or withdrawal
Anemia
Bone fracture
Cellulitis
Cholecystitis
Gastrointestinal bleed
Intestinal obstruction
Renal failure
Urinary tract infection

A CXR performed during ED evaluation prior to the admission disposition time was recorded as “pre-admission,” and, if performed after disposition time, was recorded as “admission.” A CXR was "unnecessary" if the admission diagnosis was one of the 10 listed above in Table 1. The numerator was the number of unnecessary admission CXRs ordered on patients with diagnoses unrelated to the cardiac or pulmonary systems (minus those with a pre-admission CXR); the denominator was the number of such admissions (minus those with a pre-admission CXR).

Variables of interest that might influence whether a CXR was ordered were age, gender, respiratory rate ≥20, cardiac- or pulmonary-related chief complaint, ED diagnosis category, or past medical history. The ED diagnoses were categorized as follows: allergy; back pain; dehydration; electrolyte abnormality, metabolic derangement, hyperglycemia; gastrointestinal; infectious (other than pneumonia); and neurologic.

The Chi-square test was used to compare the 2015 vs. 2016 percentages of patients with admission or pre-admission CXR. Upon finding a significant difference (p < 0.05), the Bonferroni adjusted pairwise Chi-square test was utilized to compare 2015 vs. 2016 percentages having a pre-admission CXR vs. an admission CXR.

Univariable multinomial logistic regression for each year was performed separately for each separate predictor variable, comparing pre-admission vs. admission CXR status. If a predictor variable was significantly associated with a CXR being ordered, it was entered into the multivariable model. Tukey-adjusted pairwise multiple comparisons were performed for the diagnosis categories variable. Statistical significance was determined a priori at p < 0.05. The Statistical Analysis System (SAS) version 9.4 (SAS Institute, Inc., Cary, NC, USA) was used for statistical analyses.

This project was deemed by the Institutional Review Board (IRB) to be a quality improvement project, and as such was not in need of IRB approval as it did not meet the Federal Common Rule for Human Subject Research (HSR).

## Results

From July to December 2015 there were 1,174 eligible admissions with the diagnoses listed in Table 1 that would not warrant a CXR, whereas during the same timeframe in 2016 there were 578 such admissions. Between 2015 and 2016, the hospital began encouraging disposition to home or a short-stay observation unit, rather than full admission, which likely explains the sharp decrease in eligible admissions. In 2015 the median time between admission order and CXR order was two hours and 52 minutes; in 2016, it was three hours and 47 minutes.

As demonstrated below in Table 2, among admitted patients with the diagnoses in Table 1, the percentage with pre-admission CXRs declined to a statistically-significant degree; in contrast, there was no change in the percentage of admission CXRs. 

**Table 2:** Change in percentage of patients who had CXR performed CXR: Chest X-ray, N: Number

	2015 (N = 1,174)	2016 (N = 578)	Absolute change	Relative change	P-value
No CXR (either "pre-admission" or "admission"): N (%)	307	169			
Admission CXR among patients without a pre-admission CXR: N (%) of patients	85 (21.7%)	58 (25.6%)	3.9%	18.0%	0.2678
Pre-admission CXR: N (%) of patients	782 (66.6%)	351 (60.7%)	-5.9%	-8.9%	0.0152

We performed a univariate analysis comparing 2015 vs. 2016 to investigate which of several potential risk factor variables for a CXR may have driven the decrease in pre-admission CXRs. Variables analyzed were age, gender, chief complaint category, respiratory rate <20, and cardiac- or pulmonary-related past medical history (PMH). Chief complaints were categorized as cardiac, endocrinologic (e.g., hyperglycemia), gastrointestinal, hematologic (e.g., anemia), metabolic, orthopedic, psychiatric, pulmonary, renal, and trauma. The relevant PMH were congestive heart failure, chronic obstructive pulmonary disease, lung cancer, and pneumonia. This analysis found statistically significant fewer CXRs performed in 2016 vs. 2015 among patients whose chief complaint suggested none was warranted. In contrast, patients with a pre-admission CXR in 2015 vs. 2016 were of similar average age, and a similar proportion of patients were male, with risk ratio (RR) <20, and with cardio-pulmonary-relevant PMH (Table 3).

**Table 3:** Comparison of risk factors for pre-admission CXR in 2015 vs. 2016 CXR: Chest X-ray, RR: Risk ratio, PMH: Past medical history

	2015 pre-admission CXR	2016 pre-admission CXR	Absolute change	Relative change	P-value		2015 admission CXR	2016 admission CXR	Absolute change	Relative change	P-value
Age (average)	64.5	66.4	1.9	2.9%	0.1863		62.0	64.0	2.0	3.2%	0.2713
Gender (% male)	46.0%	47.9%	1.9%	4.1%	0.5535		47.0%	53.1%	6.1%	13.0%	0.0576
CXR done despite no chief complaint suggesting CXR	71.6%	59.3%	-12.3%	-17.2%	0.0121		87.8%	89.1%	1.3%	1.5%	0.7688
RR <20	77.5%	82.1%	4.6%	5.9%	0.0906		86.7%	87.5%	0.8%	0.9%	0.8826
CXR done despite no PMH suggesting CXR warranted	85.5%	84.6%	-0.9%	-1.1%	0.8826		86.7%	92.2%	5.5%	6.3%	0.278

In multivariate analysis, risk factors for an unwarranted CXR (either pre- or post-admission) were age >40 and PMH of cardiovascular (CV) disease (e.g., myocardial infarction, atrial fibrillation), renal disease, or hyperkalemia (Table 4). 

**Table 4:** Risk factors for unwarranted CXR CXR: Chest X-ray, RR: Risk ratio, PMH: Past medical history, CV: Cardiovascular

Variable	RR	P
Age >40 years old	2.9	< 0.0001
Pre-admission CXR: PMH CV disease, renal disease, or hyperkalemia	20.3	< 0.0001
Admission CXR: PMH CV disease, renal disease, or hyperkalemia	17.6	< 0.0001

## Discussion

Up to 20% of medical diagnostic tests are unnecessary [13], indicating the scope of overutilization. Inappropriate testing increases false-positive and false-negative diagnostic errors [14,15], as well as raising costs. Although individual CXRs may not be expensive, given the large volume of testing, unnecessary testing may lead to substantial overall costs [16,17]. 

After introducing educational guidelines to decrease unnecessary admission CXRs on ED boarding patients, our ED found no decrease in admission CXRs, but there was an unintended decrease in pre-admission CXRs. The main driver of this change was decreasing unnecessary CXRs on patients whose chief complaint did not indicate one was warranted (Table 4). Other plausible variables (e.g., age, gender, PMH) that might have explained the decrease in pre-admission CXRs were not statistically significantly associated with the decrease. The selective association of chief complaint suggests the exercise of greater judgment or discrimination in the initial evaluation setting as recommended by our ED's CXR guideline not to perform a CXR on patients for whom there is "no clinical concern of cardiopulmonary disease on the basis of history or physical examination." Those exposed to the guidelines (either directly, or via diffusion) may have been responsive to the guideline's recommendation to use "history" as a gauge to determine which patients might or might not warrant a CXR. There was no significant change in our ED's triage process between the pre- and post-guideline implementation timeframes that might account for the decline in pre-admission CXR ordering. Neither nurses nor physicians operated under protocols, standing orders, or standardized procedures that would prompt them to order a CXR at triage before a more in-depth history and physical examination could be performed. In addition, there was no "upfront provider" or "provider in triage" conducting brief intake evaluations and ordering studies before patients are brought to an ED room for more-definitive evaluation and treatment. The absence of these processes in both the pre- and post-guideline periods makes it less likely the decline in pre-admission CXRs was due to a change in the initial ED triage and evaluation process. 

The association between this educational intervention and decreased pre-admission CXRs is likely due to several factors. First, all relevant specialties were involved: emergency medicine, hospitalist medicine, and radiology (by proxy, via the ACR recommendations). Second, no national representative organization recommends 'admission' CXRs (i.e., there was no conflict between organizations). Finally, while many guidelines encourage positive actions, the admission CXR guideline encouraged doing less (i.e., not to do a CXR ); it is easier to adhere to recommendations to not do something than to recommendations to take action, as taking action requires remembering to act and finding time to do so with the guideline’s details in mind.

The results of this study were counter to those of a previous study we conducted using an evidence-based guideline that found a reduction in unwarranted admission EKGs (44.1% to 27.5%) but an increase in pre-admission EKGs (41.8% to 55.1% ) [11]. This current CXR project failed to affect change in rates of ordering unwarranted admission CXRs, but was associated with a reduction in pre-admission CXRs. The EKG study may have demonstrated an increase in pre-admission EKGs owing to regulatory pressures to perform EKGs rapidly upon ED arrival. To optimize ST-elevation myocardial infarction (STEMI) management, EDs are held by the Centers for Medicare and Medicaid Services (CMS) to a door-to-EKG metric time (10 minutes) for patients presenting with chest pain or chest pain equivalent [18]. This regulatory standard encourages early, frequent EKG performance. As the population ages, and accumulates complex medical problems and vague presenting symptoms that may represent cardiac causes, an increase in unwarranted EKGs might be expected [19]. No such standard exists that would promote the performance of early CXRs.

As the results of this study were not as expected, we interviewed (individually) a total of 12 internal medicine hospitalist attending and resident physicians and physician assistants. We asked a standard question to each: “Why do providers on the internal medicine admitting service request an admission CXR for some admissions, even in the absence of non-cardiac/non-pulmonary concern?” Responses to this question clustered into four groups: 1) concern for the possibility of pulmonary edema if the patient receives inpatient intravenous (IV) fluid, particularly for cases in which moderate-to-large amounts of IV fluid are often given (e.g., cannabis hyperemesis, sepsis with urinary tract origin); 2) a desire to know the QTc interval, which many inpatient medications affect; 3) medico-legal concerns, to identify early on potential cardiac or pulmonary conditions in the event a clinically-symptomatic cardiac or pulmonary event or condition emerges during or shortly after hospitalization; 4) a desire to be comprehensive as having the patient in the hospital represents an opportunity to identify subclinical disease that can be further evaluated as an inpatient or outpatient. 

Barriers to success

Our effort to implement evidence-based CXR-ordering guidelines may not have succeeded as intended for several possible reasons. The well-documented multi-year time lag between changes in medical knowledge and wide-scale adoption [20] may be attributable to the need to pass through the four stages of the awareness-to-adherence model: 1) awareness a guideline exists, 2) agreement with the guideline, 3) adoption, and 4) adherence [21]. Often, only half of the physicians are even aware a guideline exists. On average, 10% of physicians will not agree with a given guideline. The decision to adhere is based on the guideline’s ease of use, applicability to the patient population, the extent of physician autonomy, culture/common practice in an environment, point-of-care reminders, and assessment of the legal risk of adhering to the guideline [22]. Up to 90% of physicians [23] occasionally order studies outside of evidence-based guidelines or without clear indications (i.e., practice defensive medicine) out of fear of legal consequences if they miss significant diagnoses [24]. Although evidence-based guidelines seek to detect disease (true positives) without over-testing the target population (and thereby generating excessive costs or false positives), some patient and provider risks are inherent to all guidelines. For example, cervical cancer screening occasionally occurs in persons below the U.S. Preventive Services Task Force's (USPSTF) recommended ages for Papanicolaou screening. Such medico-legal fear might have prompted admitting physicians to order admission CXRs. 

Operational factors might have also influenced decisions to order admission CXRs. Although sharing electronic health record (EHR) information can decrease duplicate testing [25], such records are not easily shared between organizations [26]. It is often simpler to order a CXR than obtain interpretations or direct visualization of CXRs from outside organizations. 

Had we utilized different approaches to guideline development and promotion, this project might have been more effective in achieving its primary aim. Most CXR orders are placed by house staff, whereas the CXR guidelines were developed by attending physicians. House staff participation in guideline development might have yielded greater buy-in or recollection. A prior study in which affected physicians developed guidelines for appropriate EKG ordering was associated with fewer EKGs ordered, even six months post-implementation [27]. When guidelines are developed by consensus, by end-users, or by persons with high perceived credibility, adherence improvements up to 40% have been demonstrated [28]. Such circumstances allow physicians a sense of ownership. In addition, had we employed a multimodal education program, rather than a single strategy (announcements), the guidelines might have been more widely adopted [27]. Passive education and information dissemination strategies (e.g., announcements) are often ineffective. Interactive strategies (e.g., in-person or technology-facilitated meetings and workshops) are more successful. We might have asked select house staff to introduce the guidelines to their peers at house staff educational conferences. In that setting, they could have utilized case studies, including questions to the audience if they would order a CXR for each case, and discussed the appropriate circumstances for ordering a CXR.

Additionally, we educated ED and Hospitalist providers regarding proper CXR ordering only once. However, new house staff begins each July. This project may have been more successful had we repeated the educational campaign in 2015, and again each July. Frequent reminders, audits, and peer review are associated with decreases in diagnostic testing [27]. Reductions in head CT imaging rates for patients with minor head injuries were accomplished when guidelines were implemented with an hour-long education session and reminders [29]. Involving service-line thought leaders in the reminder process would likely make them even more effective. Effective leadership also facilitates guideline implementation [30]. Clinical opinion leaders can utilize local networks to exert social influence and promote behavior change. Rather than review all data behind a new recommendation, providers commonly listen to guidance from key opinion peers when deciding whether to follow the guidelines. Clinical opinion leaders can diffuse criticism, identify and overcome barriers to change, and aid in operationalizing initiatives [27]. In our CXR initiative, LIJ ED and hospitalist thought leaders could have organized follow-up emails or online education activities and reminders.

In addition, ED boarding patients may deteriorate, and a post-admission CXR may be warranted; we were unable to account for such situations. Lastly, the myriad variations of clinical scenarios render it impossible for a guideline to cover every contingency, or entirely replace clinical judgment for when a CXR should be ordered for ED boarding patients.

Limitations

This study has several limitations. First, as a quality improvement project (rather than formal research), our findings cannot, and should not, be extrapolated to other settings, even those similar to ours. In addition, it was a single-site study, and there was a historical control group rather than a randomized control group as part of a randomized, controlled trial (RCT). Consequently, pre- vs. post-implementation patients may not have been similar in ways that influenced whether they had a CXR, although we attempted to control for this by incorporating likely confounders (age, gender, vital sign abnormalities, chief complaint, ED diagnosis category, or relevant PMH) in the data analysis. Conducting an RCT in this situation would have been logistically difficult, as it would have required exposing half of the providers to the intervention (the CXR-ordering guidelines) while not exposing the other half. Since physicians-in-training work with different colleagues during each ED shift, and share knowledge and practice patterns with one another, there would inevitably have been a mix on most shifts between physicians in the intervention group and those in the control group, thereby contaminating the effect of the intervention. Furthermore, some admission CXRs may have been indicated by a change in the patient's condition (e.g., unexpected dyspnea or hypoxia). Finally, we did not follow the trend in pre-admission vs. admission CXR ordering beyond one year after the intervention. It is possible there has been a reversion back to higher rates of unwarranted pre-admission CXR ordering, or perhaps, a continued trend toward lower rates. 

## Conclusions

Our interdepartmental collaboration was not associated with a change in admission CXRs on ED boarding patients but was associated with an unintended decrease in unwarranted pre-admission CXRs. This seems to have been driven by performing fewer CXRs on patients whose chief complaint did not suggest one was warranted. Organizations interested in reducing processes with little clinical value might adopt a similar program while emphasizing the lack of benefit to admitted patients through iterative educational programs for hospital admitting services. 

## Appendices

Appendix A

Guideline for Emergency Department 'Admission' Chest X-ray-Ordering  

Per the American College of Radiology (ACR), in patients for whom there is no clinical concern of cardiopulmonary disease on the basis of history or physical examination, routine 'admission' CXR is usually not appropriate. If a non-ED provider believes a CXR should be ordered on such a patient, please discuss it with the ED provider caring for the patient [2].

## References

[1] (2020). Routine Admission CXR (RACXR) - Core EM. https://coreem.net/core/routine-admission-cxr-racxr/.

[2] (2000). American College of Radiology ACR Appropriateness Criteria. https://acsearch.acr.org/docs/69451/Narrative/.

[3] United States Department of Health and Human Services (1983). Monthly Catalog of United States Government Publications: National Center for devices, radiological Health. The selection of patients for X-ray screening examinations.. Monthly Catalog of United States Government Publications: National Center for devices, radiological Health. The selection of patients for X-ray screening examinations..

[4] Malnick S, Duek G, Beilinson N (2010). Routine chest X-ray on hospital admission: does it contribute to diagnosis or treatment?. Isr Med Assoc J.

[5] Verma V, Vasudevan V, Jinnur P, Nallagatla S, Majumdar A, Arjomand F, Reminick MS (2011). The utility of routine admission chest X-ray films on patient care. Eur J Intern Med.

[6] Logan JA, Vallance R, Williams BO (1997). Does a chest x-ray alter the management of new patients attending a geriatric day hospital?. Health Bull (Edinb).

[7] Hubbell FA, Greenfield S, Tyler JL, Chetty K, Wyle FA (1985). The impact of routine admission chest x-ray films on patient care. N Engl J Med.

[8] Berrington de González A, Darby S (2004). Risk of cancer from diagnostic X-rays: estimates for the UK and 14 other countries. Lancet.

[9] Rucker G, Kalayanamitra R, Gopaul R (2021). Assessment of chest X-ray utilization for the evaluation of non-traumatic chest pain in an academic emergency department. Int J Radiol Imaging Technol.

[10] McCusker J, Vadeboncoeur A, Lévesque JF, Ciampi A, Belzile E (2014). Increases in emergency department occupancy are associated with adverse 30-day outcomes. Acad Emerg Med.

[11] Appold B, Soniega-Sherwood J, Persaud R (2021). Reining in unnecessary admission EKGs: a successful interdepartmental high-value care initiative. Cureus.

[12] Sahadeo PA, Dym AA, Berry LB (2021). The best of both worlds: eliminating creatine kinase-muscle/brain (CK-MB) testing in the emergency department leads to lower costs without missed clinical diagnoses. Cureus.

[13] Zhi M, Ding EL, Theisen-Toupal J, Whelan J, Arnaout R (2013). The landscape of inappropriate laboratory testing: a 15-year meta-analysis. PLoS One.

[14] Korenstein D, Chimonas S, Barrow B, Keyhani S, Troy A, Lipitz-Snyderman A (2018). Development of a conceptual map of negative consequences for patients of overuse of medical tests and treatments. JAMA Intern Med.

[15] Sarkar MK, Botz CM, Laposata M (2017). An assessment of overutilization and underutilization of laboratory tests by expert physicians in the evaluation of patients for bleeding and thrombotic disorders in clinical context and in real time. Diagnosis (Berl).

[16] Strauss R, Cressman A, Cheung M (2019). Major reductions in unnecessary aspartate aminotransferase and blood urea nitrogen tests with a quality improvement initiative. BMJ Qual Saf.

[17] Vegting IL, van Beneden M, Kramer MH (2012). How to save costs by reducing unnecessary testing: lean thinking in clinical practice. Eur J Intern Med.

[18] Krumholz HM, Anderson JL, Brooks NH (2006). ACC/AHA clinical performance measures for adults with ST-elevation and non-ST-elevation myocardial infarction: a report of the American College of Cardiology/American Heart Association Task Force on Performance Measures (Writing Committee to Develop Performance Measures on ST-Elevation and Non-ST-Elevation Myocardial Infarction). Circulation.

[19] Jaul E, Barron J (2017). Age-related diseases and clinical and public health implications for the 85 years old and over population. Front Public Health.

[20] Morris ZS, Wooding S, Grant J (2011). The answer is 17 years, what is the question: understanding time lags in translational research. J R Soc Med.

[21] Pathman DE, Konrad TR, Freed GL, Freeman VA, Koch GG (1996). The awareness-to-adherence model of the steps to clinical guideline compliance. The case of pediatric vaccine recommendations. Med Care.

[22] Cabana MD, Rand CS, Powe NR, Wu AW, Wilson MH, Abboud PA, Rubin HR (1999). Why don't physicians follow clinical practice guidelines? A framework for improvement. JAMA.

[23] Sekhar MS, Vyas N (2013). Defensive medicine: a bane to healthcare. Ann Med Health Sci Res.

[24] Hurwitz B (2004). How does evidence based guidance influence determinations of medical negligence?. BMJ.

[25] Lammers EJ, Adler-Milstein J, Kocher KE (2014). Does health information exchange reduce redundant imaging? Evidence from emergency departments. Med Care.

[26] (2014). Doctors Find Barriers to Sharing Digital Medical Records - The New York Times. https://www.nytimes.com/2014/10/01/business/digital-medical-records-become-common-but-sharing-remains-challenging.html.

[27] Wachtel TJ, O'Sullivan P (1990). Practice guidelines to reduce testing in the hospital. J Gen Intern Med.

[28] Prior M, Guerin M, Grimmer-Somers K (2008). The effectiveness of clinical guideline implementation strategies—a synthesis of systematic review findings. J Eval Clin Pract.

[29] Curran JA, Brehaut J, Patey AM, Osmond M, Stiell I, Grimshaw JM (2013). Understanding the Canadian adult CT head rule trial: use of the theoretical domains framework for process evaluation. Implement Sci.

[30] Sinuff T, Cook D, Giacomini M, Heyland D, Dodek P (2007). Facilitating clinician adherence to guidelines in the intensive care unit: a multicenter, qualitative study. Crit Care Med.

